# Circular HER2 RNA positive triple negative breast cancer is sensitive to Pertuzumab

**DOI:** 10.1186/s12943-020-01259-6

**Published:** 2020-09-11

**Authors:** Jie Li, Maoguang Ma, Xuesong Yang, Maolei Zhang, Jingyan Luo, Huangkai Zhou, Nunu Huang, Feizhe Xiao, Bingquan Lai, Weiming Lv, Nu Zhang

**Affiliations:** 1 Institute of Precision Medicine, The First Affiliated Hospital of Sun Yat-sen University, Guangzhou, 510080 Guangdong China; 2 Department of Breast and Thyroid Surgery, The First Affiliated Hospital of Sun Yat-sen University, Guangzhou, 510080 Guangdong China; 3 Department of Neurosurgery, Guangdong Provincial Key Laboratory of Brain Function and Disease, The First Affiliated Hospital of Sun Yat-sen University, No 58, Zhongshan 2 Road, Guangzhou, 510080 Guangdong China; 4 Forevergen Biosciences Center, R&D Unit 602, Guangzhou, 510000 China; 5 Department of Scientific Research Section, The First Affiliated Hospital of Sun Yat-sen University, Guangzhou, 510080 Guangdong China

**Keywords:** circRNA, TNBC, Circ-HER2, Pertuzumab

## Abstract

**Background:**

Triple negative breast cancer (TNBC) remains the most challenging breast cancer subtype so far. Specific therapeutic approaches have rarely achieved clinical improvements in treatment of TNBC patients and effective molecular biomarkers are largely unknown.

**Methods:**

We used paired TNBC samples and high throughput RNA sequencing to identify differentially expressed circRNAs. Sucrose gradient polysome fractionation assay, antibody and Mass spectra were used to validate active circRNA translation. The novel protein function was validated in vitro and in vivo by gain or loss of function assays. Mechanistic results were concluded by immunoprecipitation analyses and kinase activity assay.

**Results:**

Circular HER2 RNA (circ-HER2) encoded a novel protein, HER2–103. Unexpectedly, while HER2 mRNA and protein were barely detected, circ-HER2/HER2–103 was expressed in ~ 30% TNBC clinical samples. Circ-HER2/HER2–103 positive TNBC patients harbored worse overall prognosis than circ-HER2/HER2–103 negative patients. Knockdown circ-HER2 inhibited TNBC cells proliferation, invasion and tumorigenesis in vitro and in vivo, suggesting the critical role of circ-HER2/HER2–103 in TNBC tumorigenicity. Mechanistically, HER2–103 promoted homo/hetero dimerization of epidermal growth factor receptor (EGFR)/HER3, sustained AKT phosphorylation and downstream malignant phenotypes. Furthermore, HER2–103 shared most of the same amino acid sequences as HER2 CR1 domain which could be antagonized by Pertuzumab, a clinical used HER2 antibody. Pertuzumab markedly attenuated in vivo tumorigenicity of circ-HER2/HER2–103 expressing TNBC cells but showed no effects in circ-HER2/HER2–103 negative TNBC cells.

**Conclusion:**

Our results not only demonstrated that certain TNBCs were not truly ‘HER2 negative’ but also highlighted the clinical implications of Pertuzumab in circ-HER2/HER2–103 expressing TNBC patients.

## Background

Triple negative breast cancer (TNBC) is characterized by lack of estrogen receptor (ER), progesterone receptor (PR) and HER2/*neu* oncogene (HER2) [1]. Compare with hormone receptor-positive or HER2-postive breast cancers, TNBC shows a highly aggressive clinical course, with early age of onset, stronger metastatic potential, greater relapse rate and worse overall survival [2]. Although many target therapies have been tested, no significant survival benefits are proved in TNBC, and chemotherapy remains the standard of care [3]. Thus, TNBC is a disease with aggressive behavior and poor outcomes and treatment for TNBC remains an unmet need in breast cancer care.

Circular RNAs (circRNAs) are covalently closed transcripts in eukaryotes with important biological functions [4, 5]. CircRNAs have been implicated in diseases such as neurological disorders, cardiovascular diseases and cancers [6]. CircRNAs exerted their functions majorly by acting as microRNA/protein ‘sponge’ or by acting as protein scaffold [7, 8]. Recent studies have demonstrated that circRNAs not only served as prognostic markers but also promoted proliferation or metastasis of TNBC [9, 10]. However, most of these studies were supported by micro RNA sponge mechanisms, raising the hypothesis that hidden functions of circRNAs may exist in TNBC.

To date, functional peptides or proteins generated from unconventional regions, including long intergenic non-coding RNAs (LincRNAs), 5′ un-translational region (5’UTR) and circRNAs, have been adequately demonstrated [11–13]. We previously reported that open reading frame (ORF) in circRNAs driven by internal ribosomal entry site (IRES) translates functional proteins during glioblastoma tumorigenesis [14, 15]. These newly identified proteins usually played an auxiliary role to their corresponding linear counterparts, defines a fine-tune regulatory system. Although circRNAs usually downregulated in human cancers [6], certain aberrantly expressed circRNAs may provide unique opportunities to identify specific molecular targets for cancer diagnosis and treatment.

In this study, we sought to determine novel circRNAs in TNBC. We specifically described circ-HER2, a circular form of *HER2* gene, encodes HER2–103 in parts of TNBC. We then assessed HER2–103 functions in TNBC and highlighted its clinical implication. We also demonstrated that Pertuzumab effectively inhibited the tumorgenicity of HER2–103 expressing TNBC.

## Methods

### Patients and samples

All breast cancer and paired normal tissues were collected from the First Affiliated Hospital of Sun Yat-sen University. All samples were obtained with informed consent.

### Cell culture

All breast cancer cell lines and other cell lines were obtained from American Tissue Type Culture Collection (ATCC, Manassas, VA), including BT474, BT549, MDA-MB-231, MDA-MB-468, T47D, MDA-MB-453, MCF-7, MCF-10A and HEK293T. BT474 and BT549 were cultured in RPMI-1640 supplemented with 10% FBS and 1% penicillin/streptomycin. T47D were cultured in RPMI-1640 supplemented with 10% FBS and 1% penicillin/streptomycin and 0.2 Units/ml bovine insulin. MDA-MB-231, MDA-MB-468, MDA-MB-453 and HEK293 T cell lines were cultured in DMEM supplemented with 10% FBS and 1% penicillin/streptomycin. MCF-7 were cultured in MEM supplemented with 10% FBS and 1% penicillin/streptomycin and 0.01 mg/ml human recombinant insulin. All cell lines were maintained at 37 °C in a humidified atmosphere with 5% CO_2_.

### Library construction and sequencing

Total RNA was extracted using Trizol reagent kit (Invitrogen, Carlsbad, CA, USA) according to the manufacturer’s protocol. RNA quality was assessed on an Agilent 2100 Bioanalyzer (Agilent Technologies, Palo Alto, CA, USA) and checked using RNase free agarose gel electrophoresis. Next, strand-specific rRNA depleted RNA-seq library was constructed using VAHTS Total RNA-seq (H/M/R) Library Prep Kit (Vazyme, Nanjing, China). Briefly, rRNAs were removed and the retained RNAs were fragmented into short fragments by using fragmentation buffer and reverse transcribed into cDNA with random primers. Second-strand cDNA were synthesized by DNA polymerase I, RNase H, dNTP (dUTP instead of dTTP) and buffer. Next, the cDNA fragments were purified with VAHTSTM DNA Clean Beads, end repaired, poly(A) added, and ligated to Illumina sequencing adapters. Then UNG (Uracil-N-Glycosylase) was used to digest the second-strand cDNA. The digested products were purified with VAHTSTM DNA Clean Beads, PCR amplified to complete library construction. Libraries were sequenced using Illumina X10 by Gene Denovo Biotechnology Co. (Guangzhou, China). Raw reads were filtered by fastp [16] (version 0.18.0) to obtain high quality clean reads. Bowtie2 [17] (version 2.2.8) was used for mapping reads to ribosome RNA (rRNA) database. The rRNA mapped reads were then removed. The remaining reads were further used in circRNAs and host gene analysis.

### Bioinformatics procedure for circRNA expression analysis

Sequencing data was mapped to reference genome Human GRCh38 by TopHat2 [18] (version 2. 1.1). The reads that could be mapped to the genomes were discarded, and the unmapped reads were then collected for circRNA identification. 20mers from both ends of the unmapped reads were extracted and aligned to the reference genome to find unique anchor positions within splice site. Anchor reads that aligned in the reversed orientation (head-to tail) indicated circRNA splicing and then were subjected to find_circ [5] (version 1) to identify circRNAs. The anchor alignments were then extended such that the complete read aligns, and the breakpoints were flanked by GU/AG splice sites. A candidate circRNA was called if it was supported by at least two unique back spliced reads at least in one sample. To quantify circRNAs, back-spliced junction reads were scaled to RPM (Reads Per Million mapped reads). circRNAs were blasted against the circBase [19] for annotation. To identify differentially expressed circRNAs, the edgeR package (version 3.12.1) (http://www.r-project.org/) was used with general linear model. We identified circRNAs with a fold change > 2 and *P* value < 0.05 as significant differentially expressed circRNAs.

### Bioinformatics procedure for coding gene expression analysis

Sequencing data was used to analyze coding gene expression. Data was mapped to reference genome by TopHat2 (version 2.1.1), then transcripts abundances were quantified by software RSEM [20] (version 1.2.19). There were two steps for RSEM to quantify transcripts abundances. Firstly, a set of reference transcript sequences were generated and preprocessed according to transcripts (in FASTA format) and gene annotation files (in GTF format). Secondly, reads were realigned to the reference transcripts by Bowtie alignment program and the resulting alignments were used to estimate transcript abundances. The transcript expression level was normalized by using FPKM (Fragments Per Kilobase of transcript per Million mapped reads) method. Value of transcripts from the same gene were merged to obtain reads counts and expression level at gene level. Differentially expressed coding genes were also identified by the edgeR package (version 3.12.1) (http://www.r-project.org/) with general linear model and a threshold of fold change > 2 and FDR < 0.05. KEGG pathway enrichment analysis (Fisher’s Exact Test) was performed for differentially expressed coding genes [21].

### Weighted gene co-expression network analysis

Co-expression networks were constructed using WGCNA (v1.47) package in R [22].

One hundred thirty circRNA with 13,132 coding genes (Coding genes with average FPKM > 1 and expressed in at least 3 samples) were imported into WGCNA to construct co-expression modules using the automatic network construction function block wise Modules with parameters of that power was 14, TOM Type was unsigned, merge Cut Height was 0.7, min Module Size was 50. Genes were clustered into 14 modules. Intramodular connectivity (function soft Connectivity) of each gene was calculated and genes with high connectivity tended to be hub genes which might have important functions. The networks were visualized using Cytoscape_3.3.0 [23].

### RNA quantitative real-time polymerase chain reaction

Total RNA was extracted using Trizol reagent kit (Invitrogen, Carlsbad, CA, USA) according to the manufacturer’s protocol. RNA quality was assessed on an Agilent 2100 Bioanalyzer (Agilent Technologies, Palo Alto, CA, USA) and checked using RNase free agarose gel electrophoresis. Complimentary DNA was generated using a one-step iScript cDNA synthesis Kit (Vazyme) and RT-qPCR was performed using AceQ qPCR Probe Master Mix (Vazyme) and a CFX96 real-time PCR system (Bio-Rad).

### Northern blot

Approximately 20 μg of total RNA was extracted and separated by 1.2% agarose Gel. After trans-membraned and fixed, specific probes were applied at 37 °C and washed with 0.1% SDS at temperature. Data were analyzed by Quantity One or Image Lab software (Bio-Rad, Hercules, CA).

### RNase R treatments

Rnase R (Epicenter Biotechnologies, Madison, WI, USA) treatment (20 U/μl) was performed on total RNA (20 μg) at 37 °C for 15 min.

### RNA fluorescence in situ hybridization (FISH)

Fluorescence labeled oligonucleotide probes complementary to circ-HER2 junction sequences were designed using the Clone Manager suite of analysis tools. 1 × 10^4^ Cells were seeded on a coverglass-bottom confocal dish and cultured overnight. RNA FISH assay was performed using RNA FISH kit (Suzhou GenePharma Co, Ltd., Suzhou, China) according to manufacturer’s instruction. Nuclei were stained with 4,6-diamidino-2-phenylindole. Images were acquired on ZEISS LSM 880 with Airyscan (Carl Zeiss Microscopy GmbH, Jena, Germany).

### Sucrose gradient fractionation assay

Polysome were prepared in 500ul of hypotonic buffer containing 5 mM Tris-HCl (PH 7.5), 2.5 mM MgCl_2_, 1.5 mM KCl, 1 × protease inhibitor cocktail (EDTA-free), 0.5% Triton X-100, 0.1 mg/ml cycloheximide and 0.5% sodium deoxycholate. The polysome lysate were centrifuged at 16,000×*g* for 7 min at 4 °C and supernatant was collected. The 5, 10, 20, 30, 40, and 50% sucrose solutions were made and filled in ultracentrifuge tube according to the density. The polysome supernatant was loaded carefully on top of the sucrose gradient solution followed by ultracentrifuge at 28,000 rpm for 2 h at 4 °C. Then, the sucrose gradient was collected from top to bottom at 1.5 ml per tube and the UV absorbance was determined at 254 nm. In addition, the total RNA in each tube was isolated and the RNA expression of circ-HER2 in each fraction was determined by real-time PCR.

### LC-MS analysis

Proteins were separated via SDS-PAGE and subjected to digestion with sequencing-grade trypsin (Promega, Madison, WI, USA). The digested peptides were analyzed with a QExactive mass spectrometer (Thermo Fisher Scientific, Waltham, MA, USA). The fragment spectra were analyzed using the National Center for Biotechnology Information nonredundant protein database with Mascot (Matrix Science, Boston, MA, USA).

### Stable cell line generation

Lentiviral vectors expressing were co-transfected with packaging vectors psPAX2 and pMD2G (Addgene) into HEK293T cells for lentivirus production using Lipofectamine 2000 in accordance with the manufacturer’s instructions. To establish stable cell lines, the cell lines were lentivirus infected and selected with 2 μg/ml puromycin for 72 h. To generate circ-HER2 stable knockdown MDA-MB-231, MDA-MB-468, SK-BR-3 and BT474 cell lines, lentiviral-induced shRNA (GenePharma, Shanghai, China) was used according to the manufacturer’s instructions.

### Plasmids and transfection

Circ-HER2 expression plasmid was generated by chemical gene synthesis the sequence of exon3–7 of HER2, additional circulation promoter sequences were added to the 83 bp upstream and 53 bp downstream. Flag tag was separated to both sides of circ-HER2–3XFlag vector with SA and SD sequences. The plasmids were transfected with Lipofectamine 3000 (Invitrogen, Carlsbad, CA, USA) according to the manufacturer’s instructions. The plasmids details were described in Supplementary Table 1.

### Dual-luciferase reporter system

The renillaluciferase (Rluc) and the firefly luciferase (Luc) sequences were amplified from a psicheck2 vector (Promega, USA). The Rluc was placed in front and the Luc was placed in the back. The full-length sequences of Rluc-Luc were obtained by overlapping PCR and the flank sequences were connected to pCDNA 3.1(+) vector by two restriction enzyme sites NheI and XhoI. The potential IRES sequences of circ-HER2 were amplified and inserted in the middle of Rluc and Luc by two restriction enzyme sites KpnI and EcoRI introduced by primers.

### mCherry-conjugated HER2–103 live image

5 × 10^3^ MDA-MB-231 or MDA-MB-468 were seeded into 35 mm confocal dishes. HER2–103-mCherry linearized plasmid was chemically generated and transfected into MDA-MB-231 and MDA-MB-468 cells respectively with Lipofectamine 3000 according to the manufacturer’s protocol. After 48 h, the confocal dish was subjected to ZEISS LSM 880 Confocal microscope analysis (Carl Zeiss Microscopy GmbH, Jena, Germany).

### CCK8 assay

2 × 10^3^ cells were seeded into 96-well plates. 100ul medium containing 10% WST-8 regent was added to each well on days 1, 2, 3, 4 and 5. After 2 h of incubation at 37 °C, the absorbance at 450 nM was measured.

### Colony formation assay (2D)

For colony formation assay, the cells were plated in 6-well plates at a density of 2 × 10^3^/well, after two weeks incubation at 37 °C, colonies were fixed with absolute methanol and stained with 0.1% crystal violet for 20 min. Cell colonies were then examined.

### Soft-agar growth assay (3D)

Blend a total of 1 × 10^4^ stably transfected cells in prewarmed (37 °C) 0.6% soft-agar containing regular medium and pour the mixture on top of 1.2% agar in a 6-well plate. Add five drops of normal growth media per 3 days. After 2 weeks, the number and size of colonies were counted and measured in 10 random fields using ImageJ.

### Migration transwell assay

3× 10^4^ cells in 200 μL serum-free regular medium was added to the cell culture inserts with an 8-μm microporous filter (Falcon 353,097) in the upper chamber, 750 μL regular medium containing 10% FBS was added to the bottom chamber. After 48 h of incubation, the cells on the lower surface of the filter were fixed and stained for microscopic examination. Cells in five random optical fields (× 100 magnification) from triplicate filters were counted and averaged.

### Antibodies

Antibodies against EGFR (#4267), phospho-EGFR Tyr1068 (#3777), HER2 (#4290), phosphor-HER2 Tyr1221/1222 (#2243), HER3 (#12708), phosphor-HER3 Tyr1289 (#2842), pan-AKT (#4691) and phosphor-AKT Thr308 (#D25E6) were from Cell Signaling Technology (Danvers, MA, USA). Antibody against phosphor-HER2 Y1139 (ab53290) and phosphor-AKT Thr308 (ab8933) was from Abcam (Cambridge, MA, USA). Antibodies against flag (F1804) and beta-tubulin (T5201) were from Sigma-Aldrich (St. Louis, MO, USA). A polyclonal antibody against the HER2–103aa peptide produced by circ-HER2 was obtained by inoculating rabbits. The antibody was purified using affinity chromatography columns. Western blotting was performed as per standard protocols. The antibody used in this study was diluted at 1:250.

### Western blotting

Equal amount of protein lysates was resolved by SDS-PAGE gels and then transferred on a PVDF membrane (Millipore, Massachusetts, USA). After incubation with a primary antibody at 4 °C overnight, the membranes were hybridized with a secondary antibody at room temperature for 1 h. The immunoreactive signals were visualized by enhanced chemiluminescene kit.

### Co-immunoprecipitation

Cell lysate was harvested using ice-cold nondenaturing lysis buffer (Thermo Fisher Scientific). CoIP assay was performed using the Pierce Co-Immunoprecipitation Kit (Thermo Fisher Scientific) according to the manufacturer’s protocol. The corresponding antibody was first immobilized for 2 h using Amino-Link Plus coupling resin. The resin was then washed and incubated with cell lysate overnight. After incubation, the resin was again washed, and protein eluted using elution buffer.

### EGFR activity assay

Quantification of phosphorylated and total EGFR was performed with LI-COR Image Studio software. The ratio of these signal intensities (calculated as phosphorylated EGFR divided by total EGFR) at each plasmid concentration was determined and the background value from the unstimulated sample subtracted. Data were plotted as log [plasmid] versus response using GraphPad Prism, from which the maximum response for each experiment was determined. Results for each concentration were then normalized by the maximum response for the relevant experiment, and values of mean response ± SD were plotted.

### Animal studies

Four- to six-week-old female BALB/c nude mice were purchased from the Laboratory Animal Center of Sun Yat-sen University and maintained in a temperature-controlled (21 °C) and light-controlled pathogen-free animal facility with free access to food and water. All animals were treated in accordance with guidelines of the Committee on Animals of the Sun Yat-sen University. 5 × 10^6^ of MDA-MB-231 cells, 8 × 10^6^ of BT549 cells and 5 × 10^6^ of MDA-MB-468 cells were inoculated into the mammary fat pad of female BALB/c nude mice. Treatment consisted of twice weekly intraperitoneal injection of 20 mg/kg Pertuzumab (420 mg /14 ml, Roche Pharma) in PBS. Control mice were given vehicle alone. Tumors were measured once weekly with digital calipers and tumor volumes were calculated by the formula: volume = length × (width)^2^/2. For the lung metastasis model, 1 × 10^6^ cells suspended in 100 μl PBS were injected intravenously in the tail vein into 4-week-old BALB/c nude mice. Eight weeks after injection, the mice were sacrificed. The lungs were removed and fixed with phosphate-buffered formalin. Then, the numbers of metastasis nodules in the lungs were examined.

### Hematoxylin and eosin (HE) staining

Paraffin embedded mice brain was sliced (4 μM), rehydrated through a xylene and ETOH series, and then stained with Hematoxylin (Gill’s 1X) for 5 min. Rinse slides in running tap water for 5 min and dunk in Acid Alcohol (1%HCl in 70%ETOH) 2–3 times until the sections turn pink. Rinse slides in tap water again for 3–5 min and 5–6 slow dunks in ammonia Water (1 mL NH4OH in 1 L H2O). Rinse slides 3–5 min in tap water and then counterstain with Eosin Y solution for 1 min. Dehydrate through ETOH series and clear in xylene series. Coverslip slides were mounted in ProLong Gold (Invitrogen, Carlsbad, CA, USA) and left overnight at room temperature.

### Immunohistochemistry (IHC)

Tumor xenografts or surgical specimen tissue slides were deparaffinized, rehydrated through an alcohol series followed by antigen retrieval with sodium citrate buffer. Tumor sections were blocked with 5% normal goat serum (Vector) with 0.1% Triton X-100 and 3% H2O2 in PBS for 60 min at room temperature and then incubated with Anti-p-EGFR (1:400), Anti-p-AKT (1:250) overnight. Expression levels of those antigens were then detected by HRP conjugated DAB.

### Statistical analysis

Statistical analysis was carried out using Microsoft Excel 2013 and GraphPad Prism version 5.00 for Windows. Experimental data are represented as the average ± SD of a minimum of three biological replicates. Otherwise indicated, The Student’s two tailed unpaired t test was used to determine statistical significance of in vitro experiments. The log-rank test was used to determine the statistical differences of the survival data. All statistical tests were two-sided, and a *p* value of less than 0.05 was considered statistically significant.

## Results

### Characterization of Circ-HER2

**Fig. 1:**
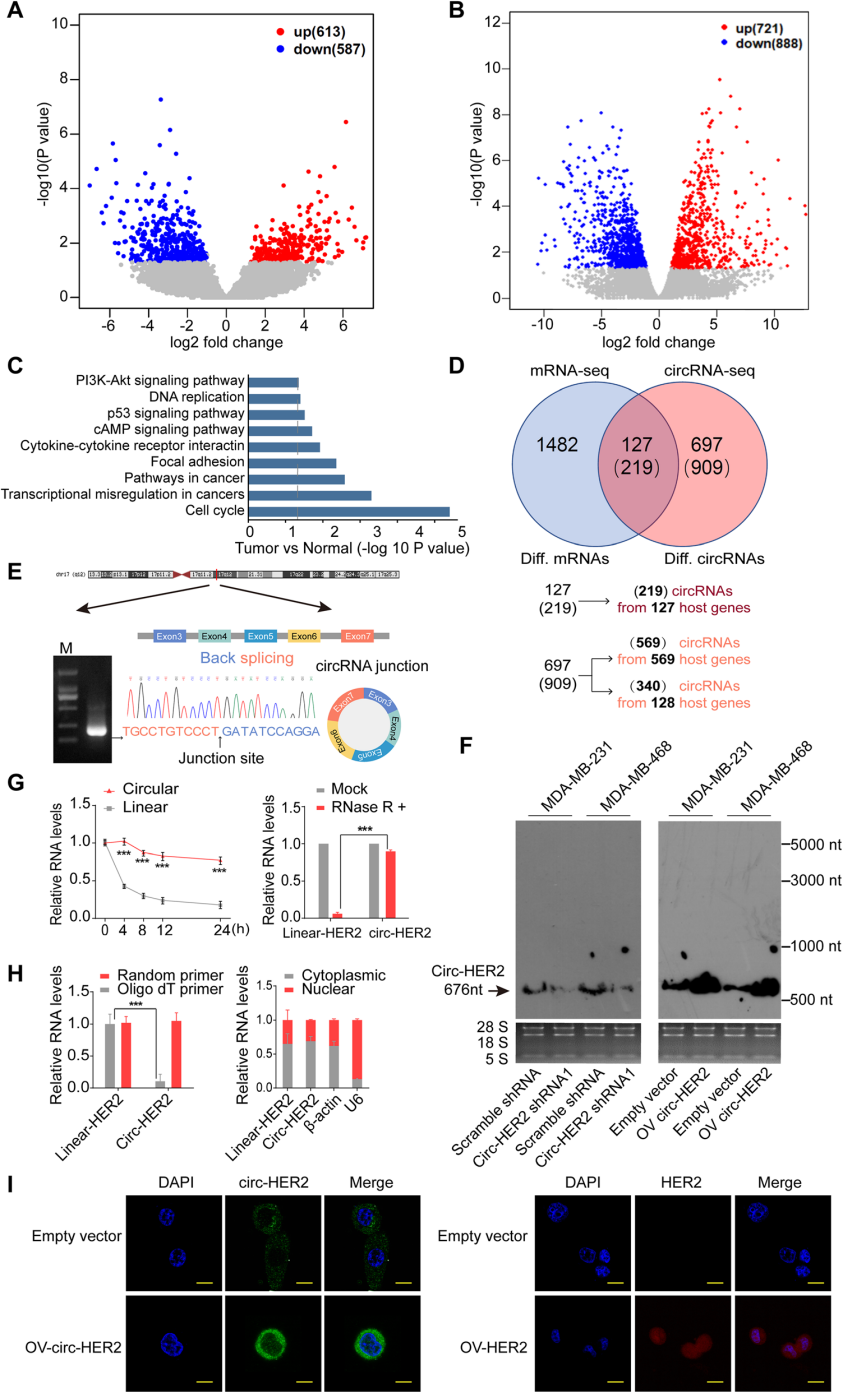
Characterization of Circ-HER2. **a** The volcano plot of differentially expressed circRNAs between TNBC and tumor adjacent normal tissues (*n* = 5). X axis, −log10 *P* value; Y aixs, log2 fold change. **b** The volcano plot of differentially expressed host coding gene between TNBC and tumor adjacent normal tissues (*n* = 5). X axis, −log10 P value; Y aixs, log2 fold change. **c** KEGG pathways enriched among differentially expressed host coding genes. **d** Comparison of differentially expressed host coding genes and differentially expressed circRNAs. Numbers in brackets in Venn diagram represent the number of differentially expressed circRNAs derived from corresponding host coding genes. **e** Illustration of the annotated genomic region of *HER2* gene, the putative different mRNA splicing forms (linear splicing and ‘head-to-tail’ splicing) and the validation strategy for the circular exon 3–7 (circ-HER2). Sanger sequencing following PCR using the indicated divergent flanking primers showed the ‘head-to-tail’ splicing of circ-HER2 in MDA-MB-231 cells. **f** Northern blots using the junction-specific probe to detect circ-HER2 in MDA-MB-231 and MDA-MB-468 cells endogenously or with indicated modifications. **g** Left, relative RNA level of circ-HER2 and linear HER2 at different time point; Right, relative RNA level of circ-HER2 and linear HER2 treated with RNase R. **h** Left, total RNA from MDA-MB-231 cells were reverse-transcribed with Oligo dT primers or random primers; circ-HER2 or linear HER2 mRNA levels were detected by q-PCR. Right, cytoplasmic and nuclear fractions were isolated, circ-HER2 and linear HER2 expression were detected. β-actin and U6 RNA served as cytoplasmic and nuclear RNA markers. **i** Fluorescence in situ hybridization (FISH) with junction-specific probes (green) or linear specific probes (red) were used to detect the localization of circ-HER2 and HER2 in MDA-MB-231 TNBC cells. Circ-HER2 and linear HER2 plasmids were transfected as positive controls. Scale bars, 20 μm. Lines show the mean ± SD. ***p < 0.001. In (E), (F), (G), (H), (I), Data are representative from at least 2–3 experiments with similar results

To define TNBC, ER and PR expression should less than 1% of tumor cells nuclei by immunohistochemistry (IHC), while HER2 expression is initially assessed by IHC with lack of overexpression defined as IHC scores 0 or 1 [24, 25]. We used five clinically defined TNBC samples and their paired normal tissues to perform high-throughput circRNA sequencing as previously described [26]. A total of 27,346 circRNAs were identified and 1200 were differentially expressed between TNBC cancerous and paired normal tissues (*p* < 0.01 and fold change > 2; 613 up regulated in TNBC and 587 down regulated in TNBC; Supplementary Table 2). Of these differential circRNAs, 1128 were generated from 824 host coding genes and 72 were derived from intergenic regions (Fig. 1a). We also analyzed differentially expressed host coding genes (RNA-seq) from these paired samples. A total of 1609 differential coding genes were identified (*p* < 0.01 and fold change > 2; 721up regulated in TNBC and 888 down regulated in TNBC, Fig. 1b; Supplementary Table 3). KEGG (Kyoto Encyclopedia of Genes and Genomes) analyses indicated that these differentially expressed coding genes were enriched in signals such as ‘PI3K-Akt pathway, Focal adhesion, Transcriptional mis-regulation in cancers’, which was consistent with those most significantly activated signaling pathways in TNBC [27] (Fig. 1c). We further cross-matched the circRNA-seq and RNA-seq data described above. One hundred twenty-seven host coding genes’ mRNA and 219 circRNAs derived from them were both differentially expressed between TNBC and normal tissues. One thousand four hundred eighty-two host coding genes were differentially expressed in mRNA level, but not in circRNA level. mRNA of the rest 697 host coding genes (909 differentially expressed circRNAs were derived them) were not differentially expressed between paired samples (Fig. 1d). We specifically focused on these 909 circRNAs, as they may exert independent functions from their host coding genes’ linear products. To narrow down targets, we focused on those circRNAs had more than 10 junction reads and with circBase annotation and 130 candidates were selected. We next performed combined weighted gene correlation network analysis (WGCNA) of these 130 circRNAs and 13,132 coding genes (FPKM> 1 and at least expressed in 3 samples; Supplementary Figure S1A-D). Fourteen modules were enriched, and the darkred module were genes that highly expressed in TNBC than normal tissues (Supplementary Figure S1E). This module contained 1425 host genes and 12 circRNAs (Supplementary Table 4). At least 10 breast cancer related hub genes were ranked top 15% in this module, and 5 circRNAs were highly related to these 10 hub genes (weight value> 0.15, Supplementary Figure 1F). Circ-HER2 (hsa_circ_0007766) was among these 5 narrow-down candidates. Specifically, HER2 was lowly expressed in TNBC compared with normal tissues (log2FC = − 0.87, FDR > 0.05), while circ-HER2 was overexpressed in TNBC (log2FC = 1.74, *P* < 0.05).

Inspection of HER2 genomic region revealed that circ-HER2 was formed from exon 3–7 of *HER2* gene (Fig. 1e, upper). By designing divergent primers and following sanger sequencing, we confirmed the predicted circular junction of circ-HER2 [19] in MDA-MB-231 TNBC cells (Fig. 1e, lower). To confirm circ-HER2 expression, we designed junction probe and performed northern blot in MDA-MB-231, MDA-MB-468 cells expressing circ-HER2 junction shRNA or circ-HER2 vector. Junction probe detected endogenous circ-HER2 in both cell lines. Circ-HER2 junction shRNA (referred as sh-1) and overexpression (OV) of circ-HER2 expression vector decreased or upregulated circ-HER2 expression in both cell lines, suggested the specificity (Fig. 1f). qPCR results also showed that compare with HER2 mRNA, circ-HER2 presented a longer half-life (Fig. 1g, left) and was more resistant to RNase R digestion (Fig. 1 g, right). Using junction specific primers, we only amplified circ-HER2 in random primer reverse-transcripted but not in oligo dT reverse-transcripted cDNA from MDA-MB-231 cells (Fig. 1h, left). Cell fraction qPCR in MDA-MB-231 cells (Fig. 1 h, right) and fluorescence in situ hybridization (FISH) (Fig. 1i, left) identified that most circ-HER2 expressed in cytoplasm, which was inconsistent to a previous report [28]. Besides, circ-HER2 and linear HER2 mRNA had different FISH signals in MDA-MB-231 TNBC cells (Fig. 1i, right).

### Circ-HER2 is enriched in part of TNBC

**Fig. 2:**
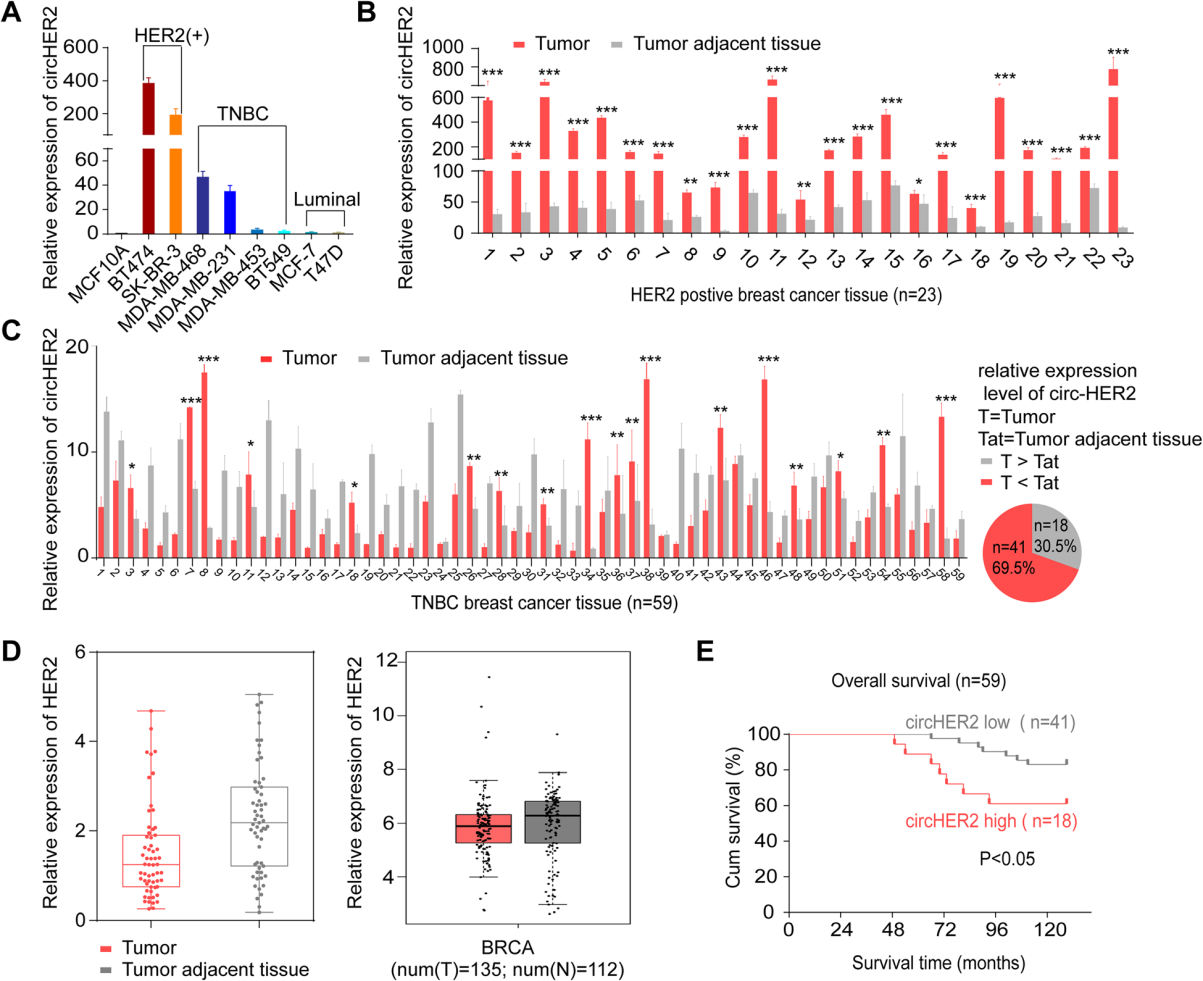
Circ-HER2 is enriched in part of TNBC. **a** The relative expression levels of circ-HER2 determined by q-PCR using junction specific primers in mammary normal cell lines (MCF10A) and different types of breast cancer cell lines as indicated. **b** The relative expression levels of circ-HER2 in 23 paired HER2 positive breast cancer specimens and the corresponding paired normal adjacent tissues was determined by using junction specific primers. **c** Left, the relative expression levels of circ-HER2 in 59 paired TNBC specimens and the corresponding paired normal adjacent tissues was determined by using junction specific primers; right, the ratio of circ-HER2 enrichment in 59 TNBC tissues. **d** Left, the relative expression levels of linear HER2 in 59 paired TNBC specimens and the corresponding paired normal adjacent tissues was determined by using linear specific primers. Right, the relative expression levels of linear HER2 in TNBC tissues from TCGA database. **e** Kaplan-Meier analysis of the correlation between circ-HER2 RNA expression and overall survival in the 59 TNBC patient’s cohort. Lines show the mean ± SD, ***, *p* < 0.001; **, *p* < 0.01; *p < 0.05. Data are representative from at least 2–3 experiments with similar results

CircRNAs usually shared with the same expression pattern with their cognate linear mRNA [6]. We found that HER2-postive tumor cells BT474 and SK-BR-3 expressed considerably higher circ-HER2 compared with MCF10A. Circ-HER2 was also overexpressed in MDA-MB-468, MDA-MB-231, but not in MDA-MB-453 and BT549 TNBC cells. Luminal type tumors, such as MCF7 and T47D, had comparable circ-HER2 expression to MCF10A (Fig. 2a). In a cohort of 23 HER2-positive breast cancer patients, considerably higher circ-HER2 expression was identified in all cancerous samples compared with that in adjacent normal tissues (Fig. 2b). In another cohort of 59 TNBC patients, 30.5% (18 out of 59) TNBC samples harbored higher circ-HER2 expression than adjacent normal tissues (Fig. 2c). The average Ct value of circ-HER2 in 18 patients was 25.5 (internal control β-actin was 23), suggesting that circ-HER2 was not a lowly expressed circRNA in these TNBC. These 59 cancerous samples had the same expression level of HER2 mRNA when comparing adjacent normal tissues, which was consistent with TCGA database (Fig. 2d). We next performed survival analysis of these TNBC patients with higher circ-HER2 expression (tumor > adjacent normal) compare with those with lower circ-HER2 expression patients (adjacent normal > tumor). Circ-HER2 status negatively correlated with overall survival time (Fig. 2e). The divergence of circ-HER2 expression compared with that of linear HER2 in TNBC and its prognostic value in TNBC promoted us for next step investigation.

### Circ-HER2 encodes HER2–103

**Fig. 3:**
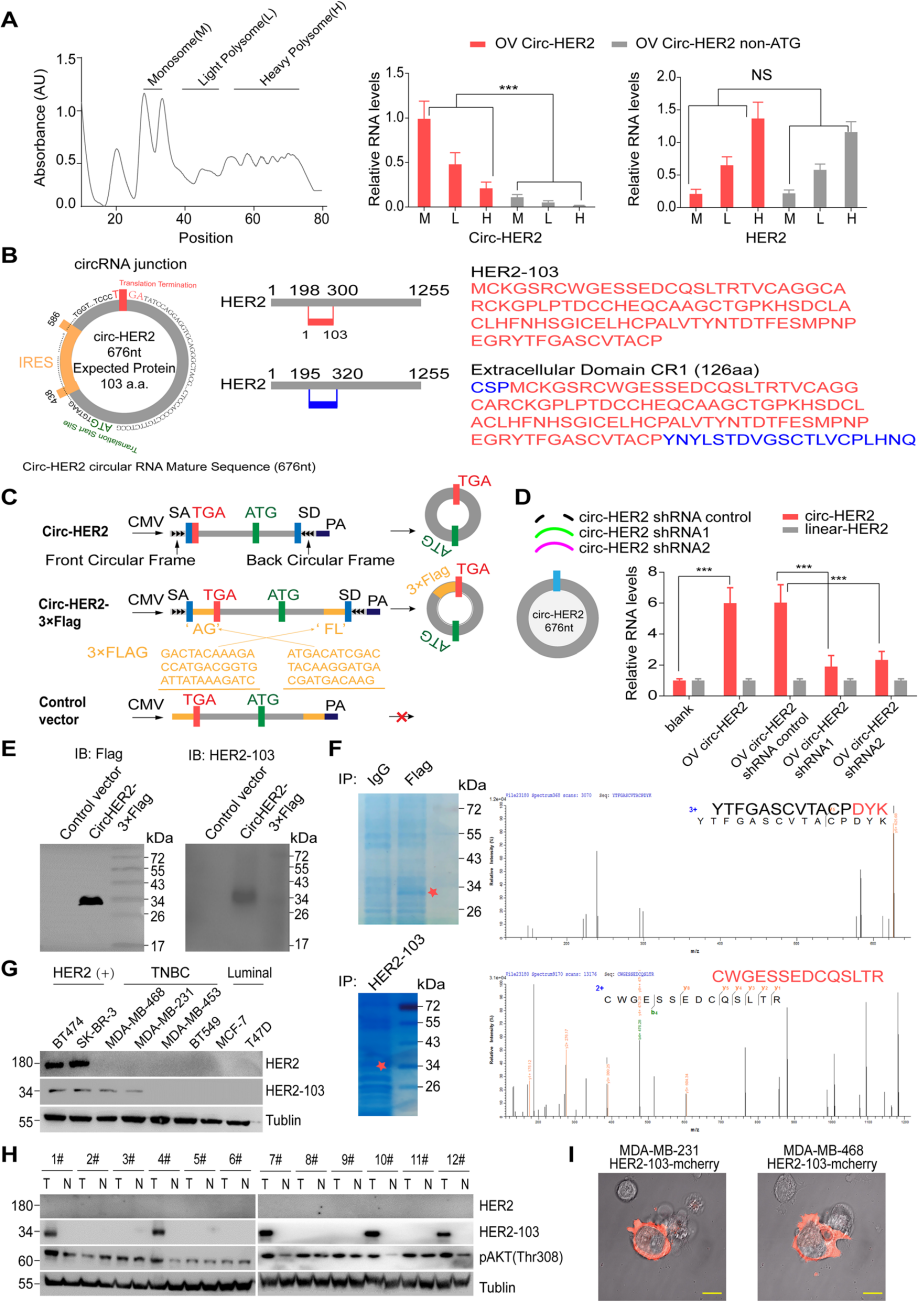
Circ-HER2 encodes a 103aa novel protein HER2–103. **a** Polysome fractions (M, monosome; L, light polysome; H, heavy polysome) in circ-HER2 and circ-HER non-ATC overexpressed 293 T cells were extracted by 5 to 50% sucrose gradient ultracentrifuge. q-PCR using junction specific primers was then performed to analyze the translation potential of circ-HER2. q-PCR using linear specific primers to determine linear HER2’s translation potential was served as positive control. **b** Left, the putative ORF, IRES and start/stop sites of circ-HER2; Right upper, illustration of HER2–103 amino acid sequence. Right lower, illustration of the extracellular CR I domain amino acid sequences HER2. Different amino acid sequences of CR I domain and HER2–103 was shown in blue. **c** Illustration of the synthetic circRNA expression plasmid: circ-HER2, exon 3–7 sequences of the HER2 gene were cloned between SA and SD with both sides of flanking repeat sequences (front circular frame and back circular frame); circ-HER2–3 × Flag, exon 3–7 sequences of the HER2 gene were cloned between SA and SD with both sides of flanking repeat sequences as indicated, with 3XFlag tag sequences were separately cloned at both sides; control vector, SA, SD and flanking repeat sequences were removed from circ-HER2–3 × Flag vector to prevent circularization. **d** Left, circ-HER2 overexpression plasmid as well as two circ-HER2 junction shRNAs and a control shRNA were designed, transfected into HEK293T cells as indicated. Right, relative circ-HER2 and linear HER2 RNA levels were detected by q-PCR using junction specific or linear specific primers. **e** Total proteins from circ-HER2–3 × Flag or control plasmid-transfected HEK239T cells were prepared, and HER2–103 overexpression was confirmed by immunoblotting using Flag antibody or HER2–103 antibody. **f** Upper, total proteins from circ-HER2–3 × Flag or control plasmid-transfected HEK239T cells were separated via SDS-PAGE. The gel bands between 26 kD and 43 kD were cut and subjected to LC-MS/MS. The identified Flag-tag amino acids are shown in red; Lower, total proteins from MDA-MB-231 cells were separated via SDS-PAGE. The gel bands between 26 kD and 43 kD were cut and subjected to LC-MS/MS. The identified HER2–103 amino acids were shown in red. **g** HER2–103 and HER2 were detected by immunoblotting in eight different breast cancer cell lines as indicated. **h** HER2–103 and HER2 were detected in 12 paired TNBC samples. **i** HER2–103-mCherry plasmid was transfected to MDA-MB-468 and MDA-MB-231 cells for 72 h, confocal microscope live image was used to show the HER2–103 cellular localization. Scale bars, 20 μm. Lines show the mean ± SD, ***, *p* < 0.001; **, *p* < 0.01; *p < 0.05. Data are representative from at least 2–3 experiments with similar results

Adequate evidences had been shown that circRNAs could serve as protein or peptide template [14, 29]. We used sucrose density gradient centrifugation based polysome analysis to test that whether circ-HER2 was translated. We transfected 293 T cells with circ-HER2 or circ-HER2 ATG deletion (non-ATG) vector and performed qPCR in monosome (M), light polysome (L) and heavy polysome (H) fractions. Circ-HER2 was detected in M and L fractions, but not circ-HER2 non-ATG. As a positive control, linear HER-2 was detected mostly in L and H fractions and was not affected by circ-HER2 transfection (Fig. 3a). Inspection of circ-HER2 sequences reveled an open reading frame (ORF) in circ-HER2 which potentially generated a 103 amino acid protein (referred as HER2–103 hereafter, Fig. 3b). This ORF was driven by an internal ribosome entry site (IRES), of which the activity was validate by circular vector-based luciferase assay (Supplementary Figure 2A). HER2–103 covered most amino acid sequences of HER2 CR1 out-membrane domain. We next generated several vectors to validate circ-HER2 translation. In addition to previously used circ-HER2 vector (Fig. 3c, upper), we also designed a circ-HER2–3XFlag vector in which 3XFlag-tag was separated and inserted reversely at both sides of the predicted HER2–103 ORF (Fig. 3c, middle). The control vector which could not be circularized due to the lack of circularization elements was used as negative control (Fig. 3c, lower). Transfection of circ-HER2 vector in 293 T cells elevated circ-HER2 expression without affecting HER2 mRNA, and two circ-HER2 shRNA (sh-1 and sh-2) successfully decreased circ-HER2 level, as determined by qPCR (Fig. 3d). 3XFlag-circ-HER2 could translate a ~ 34kD Flag-tag protein in 293 T cells (Fig. 3e, left), suggested the translation of circ-HER2. We next generated an anti-HER2–103 antibody, which detected a similar single band in circ-HER2–3XFlag transfected 293 T cells but not in control vector transfected cells, suggesting the specificity (Fig. 3e, right). By performed immunoprecipitation-Mass spectra (IP-MS) using anti-Flag antibody in 293 T cells transfected with circ-HER2–3XFlag vector, we identified Flag-tag sequences around ~34kD (Fig. 3f, upper). Furthermore, by IP-MS using HER2–103 antibody, we also identified HER2–103 amino acid sequences at the predicted molecular weight in MDA-MB-231 cells, indicating the endogenous existence of HER2–103 (Fig. 3 f, lower). Using this antibody, we detected HER2–103 expression in several established breast cancer cell lines, including HER2 positive BT474, SK-BR-3, TNBC MDA-MB-468, MDA-MB-231, MDA-MB-453, BT549, and luminal T47D, MCF7 (Fig. 3g). All HER2 positive cell lines expressed HER-103. Part of TNBC cell lines also expressed HER2–103, while luminal cell lines showed negative HER2–103 expression. Notably, overexpression of HER2 in MDA-MB-453 cells did not promote circ-HER2/HER2–103 expression, excluded that circ-HER2 was positively regulated by HER2 (Supplementary Figure 2B). In clinical samples, 41% (5 out of 12) randomly selected TNBC cancerous tissues expressed HER-103 and no detectable HER2–103 was found in paired normal breast tissues (Fig. 3h). Using mCherry-conjugated HER2–103 live image experiment, we found HER2–103 mainly expressed near cell membrane (Fig. 3i). Supernatant IB also supported that HER2–103, instead of circ-HER2, was secreted from cells (Supplementary Figure 2C). Above results indicated that circ-HER2 encoded a novel HER2 variant HER2–103 which was expressed in some TNBCs.

### HER2–103 is critical for TNBC cells proliferation and invasion

**Fig. 4:**
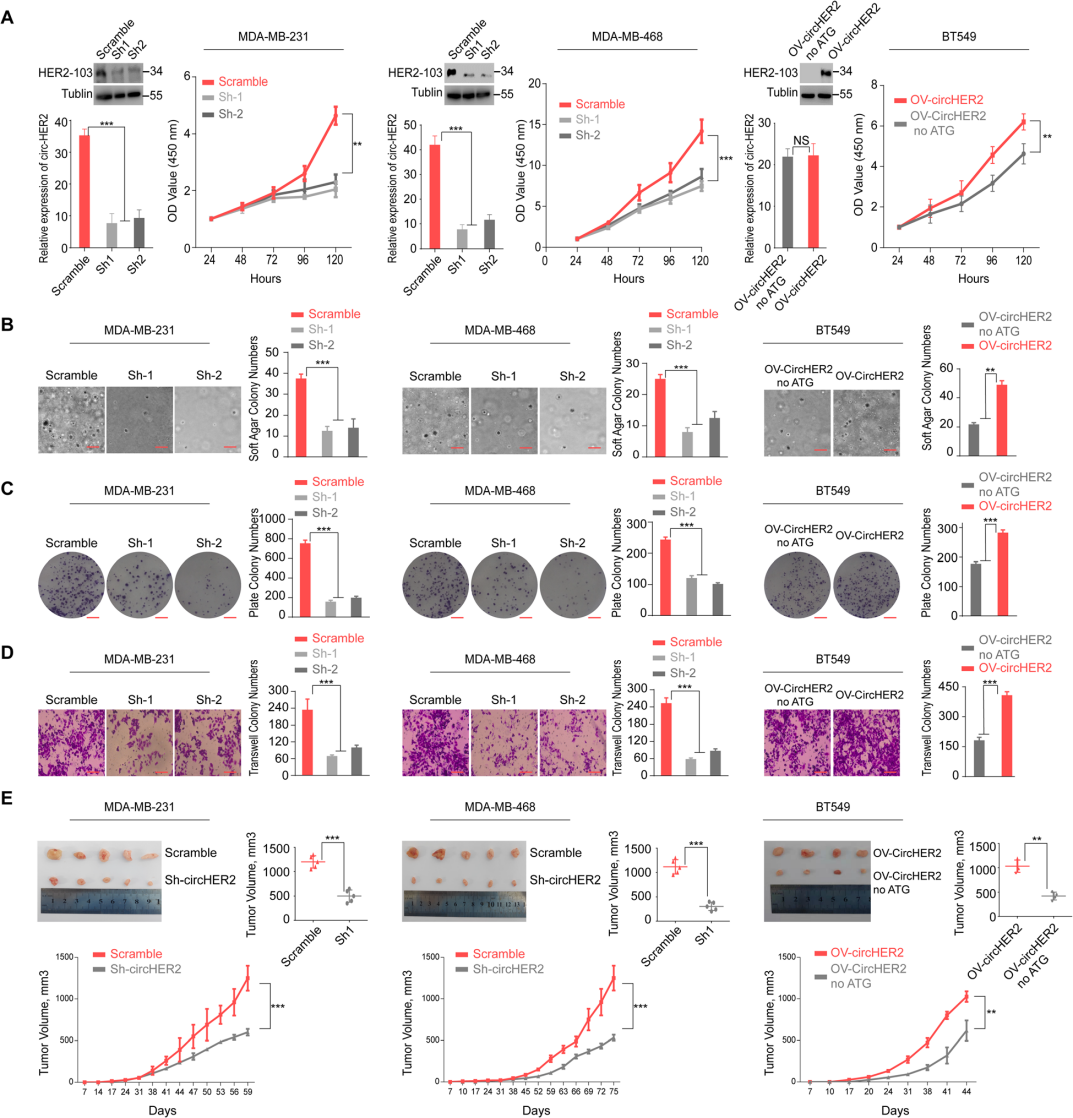
HER2–103 is critical for TNBC cells proliferation and invasion. **a** Left two panels, MDA-MB-231, MDA-MB-468 cells were stably transfected with scramble or circ-HER2 shRNAs. Circ-HER2 and HER2–103 were decided by immunoblotting and q-PCR. CCK8 cell proliferation assay was performed in above mentioned modified cells. Right, BT549 cells were stably transfected with circ-HER2 noATG vector or circ-HER2 vector. Circ-HER2 and HER2–103 were decided by immunoblotting and q-PCR. CCK8 cell proliferation assay was performed in above mentioned modified cells. **b** Left two panels, soft agar colony formation assay (3D) in MDA-MB-231 and MDA-MB-468 cells stably transfected with scramble or circ-HER2 shRNAs. Right, soft agar colony formation assay (3D) in BT549 cells transfected with circ-HER2 or circ-HER2 noATG vector. **c** Left two panels, plate colony formation assay (2D) in MDA-MB-231 and MDA-MB-468 cells stably transfected with scramble or circ-HER2 shRNAs. Right, plate colony formation assay (2D) in BT549 cells transfected with circ-HER2 or circ-HER2 noATG vector. **d** Left two panels, transwell migration assays of MDA-MB-231 and MDA-MB-468 cells stably transfected with circ-HER2 shRNAs or scramble shRNA. Right, transwell migration assays of BT549 cells transfected with circ-HER2 or circ-HER2 noATG vector. **e** Left two panels, representative tumor images and in vivo tumor growth curve in the mouse xenograft model implanted with MDA-MB-231 and MDA-MB-468 cells stably transfected with scramble or circ-HER2 shRNAs. Right, representative tumor images and in vivo tumor growth curve in the mouse xenograft model implanted with BT549 cells transfected with circ-HER2 or circ-HER2 noATG vector. Tumor volumes were calculated by: volume = length× (width)^2^/2. Lines show the mean ± SD, ***, *p* < 0.001; **, *p* < 0.01; *p < 0.05. Data are representative from at least 2–3 experiments with similar results. *N* = 5 mice in each in vivo experiment

To investigate circ-HER2/HER2–103 function in TNBC, we generated MDA-MB-231 and MDA-MB-468 HER2–103 stable knocking down (KD) cells by using sh-1 and sh-2 as previously described. Reversely, we generated HER2–103 OV BT549 cells by stably transfection of circ-HER2, with the ATG mutant circ-HER2 (non-ATG) as negative control (Fig. 4a). Modification of circ-HER2 expression altered HER2–103 level in above cells, except non-ATG circ-HER2, further supported that circ-HER2 translated HER2–103. Functionally, HER2–103 deprivation attenuated cell proliferation in both MDA-MB-231 and MDA-MB-468 cells, while overexpression HER2–103 in BT549 cells promoted cellular growth (Fig. 4a). HER-103 also positively correlated with the 3D soft agar colony formation and 2D plate colony formation in all three TNBC cell lines (Fig. 4b, c). Migration ability, which is a hallmark of metastasis for TNBC cells, also positively regulated by HER2–103 in all three cell lines (Fig. 4d). In subcutaneous xenograft model, HER2–103 deprivation inhibited tumor growth in vivo in both MDA-MB-231 and MDA-MB-468 cells. In contrast, HER2–103 OV enhanced tumorigenicity of BT549 cells (Fig. 4e). These data collectively indicated that HER2–103 positively correlated TNBC cells tumorigenicity.

### HER2–103 promoted EGFR/HER3 interaction and activation in TNBC

**Fig. 5:**
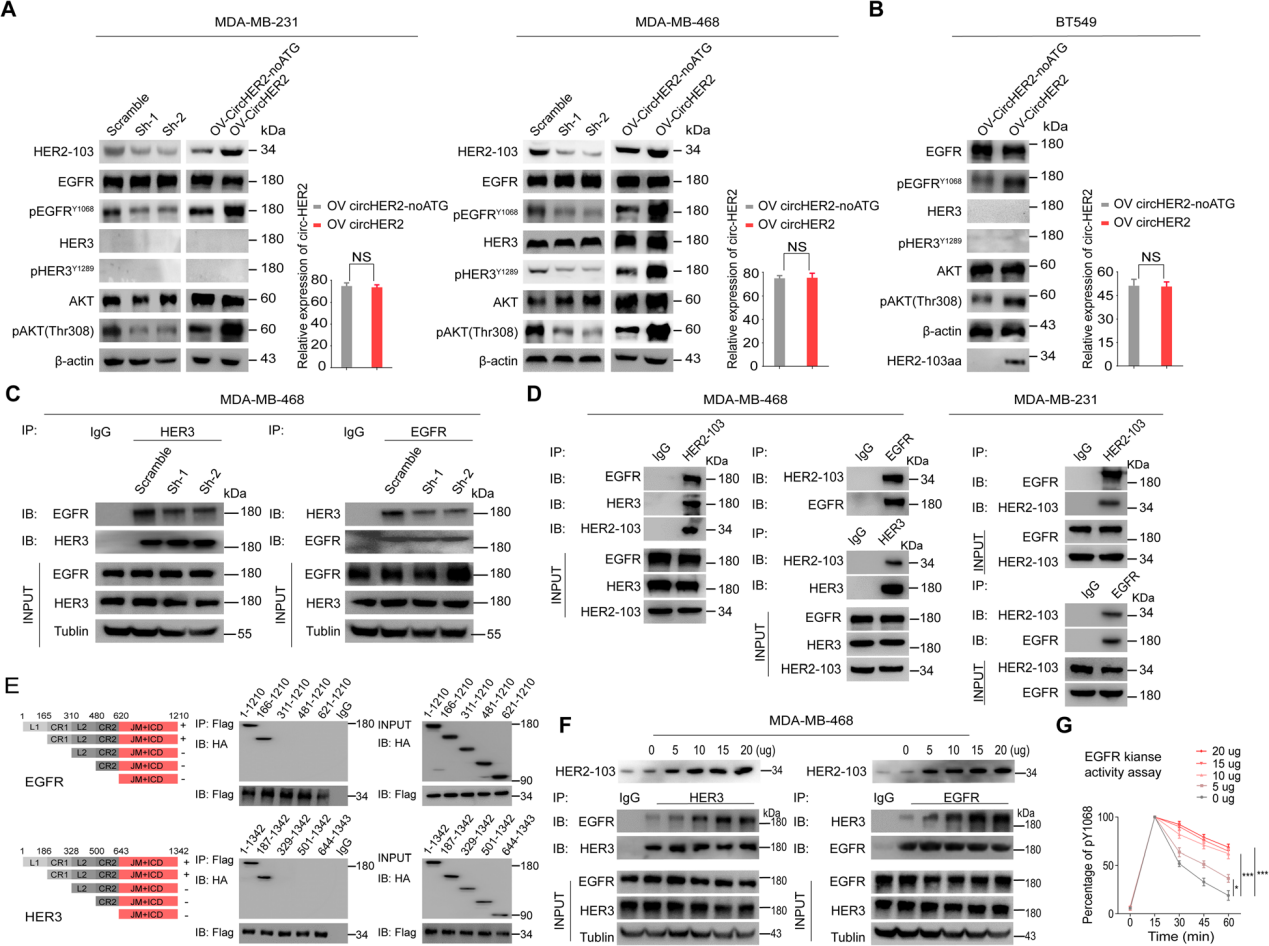
HER2–103 promoted EGFR/HER3 interaction and activation in TNBC. **a** Circ-HER2 shRNAs or scramble shRNA stably transfected MDA-MB-231 or MDA-MB-468 cells, as well as circ-HER2 and circ-HER2 noATG vector stably transfected MDA-MB-231 or MDA-MB-468 cells were subjected to immunoblotting with indicated antibodies. The transfection efficiency of above cells was verified by q-PCR. **b** circ-HER2 or circ-HER2 noATG vector stably transfected BT549 cells were subjected to immunoblotting with indicated antibodies. The transfection efficiency was verified both by q-PCR. **c** Immunoprecipitation using HER3 or EGFR antibodies were performed in circ-HER2 shRNAs or scramble shRNA stably transfected MDA-MB-468 cells with indicated antibodies. **d** Left, circ-HER2 was stably transfected to MDA-MB-468 cells, HER2–103 was immunoprecipitated, followed by immunoblotting with antibodies against EGFR and HER3; middle, EGFR and HER3 were immunoprecipitated in above mentioned cells, followed by immunoblotting with antibodies against HER2–103. Right, circ-HER2 was stably transfected to MDA-MB-231 cells, HER2–103 or EGFR were immunoprecipitated followed by immunoblotting with antibodies against EGFR and HER2–103. **e** Upper left, EGFR domains, namely, L1, CR1, L2, CR2, juxta-membrane (JM) segment and internal cellular domain (ICD). Upper right, HA-tagged EGFR domains and Flag-tagged HER2–103 were co-transfected into 293 T cells. Coimmunoprecipitated truncated EGFR protein was detected by anti-HA antibody after immunoprecipitation with anti-Flag antibody. Lower left, HER3 domains, namely, L1, CR1, L2, CR2, juxta-membrane (JM) segment and internal cellular domain (ICD). HA-tagged HER3 domains and Flag tagged HER2–103 were co-transfected into HEK293T cells. Coimmunoprecipitated truncated HER3 protein was detected by anti-HA antibody after immunoprecipitation with anti-Flag antibody. **f** HER2–103 was dose-dependently transfected into MDA-MB-468 cells. Interaction between EGFR/HER3 was determined mutually by immunoprecipitation, with indicated antibodies. **g** The kinase activity of EGFR pY1068 was detected following circ-HER2 stably transfection in MDA-MB-468 cells. Lines show the mean ± SD, ***, *p* < 0.001; **, *p* < 0.01; *p < 0.05. Data are representative from at least 2–3 experiments with similar results

Compensatory signaling, such as EGFR and HER3, activated downstream AKT to maintain malignant phenotypes in TNBC [1]. Combined EGFR/HER3 score was shown to provide prognostic and predictive significance in TNBC [30]. In TNBC cells, we found that HER2–103 KD in both MDA-MB-231 and MDA-MB-468 cells decreased EGFR or EGFR/HER3 phosphorylation respectively without affecting EGFR/HER3 protein level. Consequently, p-AKT (S308) level was also inhibited (Fig. 5a). In contrast, HER2–103 OV in BT549 cells enhanced EGFR activation and downstream p-AKT(S308) level (Fig. 5b). These results promoted us to further investigate the effects of HER2–103 on EGFR/HER3 signaling.

Without HER2, EGFR could form EGFR/EGFR homodimer or heterodimer with HER3 to activate downstream signaling cascade [31, 32]. In MDA-MB-468 TNBC cells with stable circ-HER2 KD, we found that EGFR/HER3 heterodimer formation was inhibited compare with that in control cells (Fig. 5c). We next investigated whether HER2–103 could bind to EGFR/HER3. As expected, the mutual interaction among HER2–103, EGFR and HER3 was verified in MDA-MB-468 endogenously. In MDA-MB-231 cells which did not express HER3, HER-103 and EGFR mutual interaction was detected (Fig. 5d). HER2–103 sequence covered the CR I domain of HER2, which was responsible for EGFR/HER2–103 or HER3/HER2–103 dimerization. To further explore the binding domain of HER2–103 to EGFR/HER3, we generated several truncated constructions as shown in Fig. 5e, left. IP experiment indicated that HER2–103 interacted with CR I domain (amino acid 165–310 of EGFR, amino acid 166–328 of HER3) of EGFR/HER3 (Fig. 5e, right). These results indicated that HER2–103 could induce EGFR/HER3 heterodimer or EGFR/EGFR homodimer formation by direct interaction. In MDA-MB-468 TNBC cells, dose-dependently OV HER2–103 promoted EGFR/HER3 mutual interaction (Fig. 5f), as well as EGFR kinase activity (Fig. 5g), suggesting HER2–103 could enhance EGFR/HER3 signaling cascades.

As CR I domain of HER2 also shared similar amino acid sequences as EGFR and HER3, we also explored whether HER2–103 maintained tumorigenicity in HER2 positive cells. We stably knocking down circ-HER2 in BT474 and SK-BR-3 cells (Supplementary Figure 3A). Abolishing HER2–103 also inhibited cell proliferation, colony formation and migration ability of these cells (Supplementary Figure 3B-E). Circ-HER2 KD prevented p-HER2 and AKT activation in both cells, indicated HER2–103 was essential for HER2 activation (Supplementary Figure 3A). HER2–103 also interacted with HER2 CR I domain, suggested HER2–103 could promoted HER1-HER3 dimerization and activation (Supplementary Figure 4A, B).

### HER2–103 expressing TNBC are sensitive to Pertuzumab

**Fig. 6:**
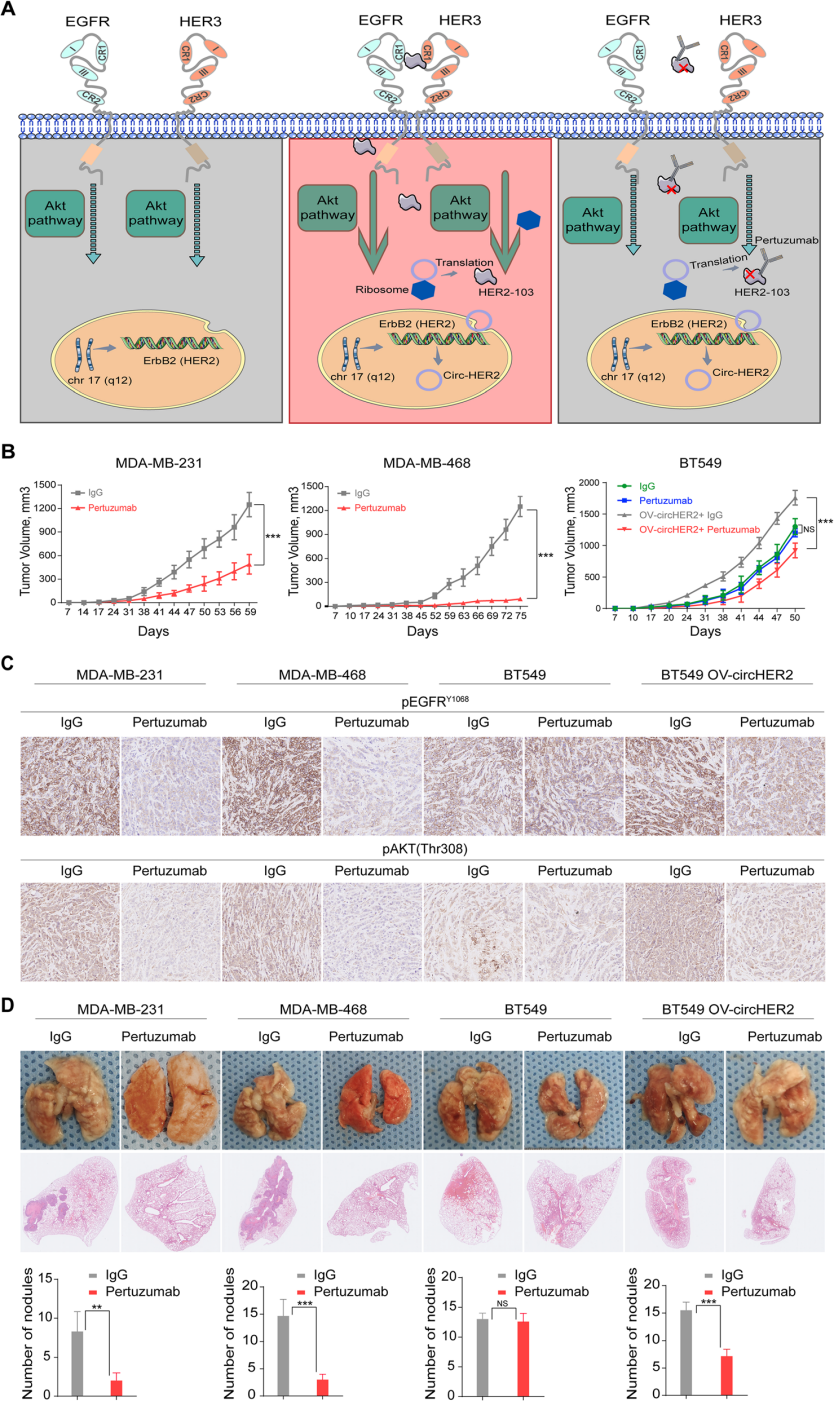
HER2–103 expressing TNBC are sensitive to Pertuzumab. **a** Illustration of circ-HER2 mechanisms in TNBC. Circ-HER2 encoded HER2–103 in TNBC cells. HER2–103 induced EGFR/EGFR homodimer and/or EGFR/HER3 heterodimer, activated downstream PI3K-AKT pathway and promoted tumor proliferation and progression. This oncogenic phenotype induced by HER2–103 can be inhibited by Pertuzumab, which recognize CR I domain of HER2. **b** Tumor volume of in vivo xenograft implanted with indicated TNBCs and treated with control IgG or Pertuzumab (20 mg/Kg). **c** Representative IHC staining of pEGFR^Y1068^ and pAKT (Thr308) in each xenograft group as indicated. **d** Representative lung and representative HE staining of lung metastatic lesions in each xenograft group as indicated, original magnification, × 4, scale bar, 100 μm. The number of metastatic nodules formed in the lungs of mice for each group were shown. Lines show the mean ± SD, ***, *p* < 0.001; **, *p* < 0.01; *p < 0.05. Data are representative from at least 2–3 experiments with similar results. *N* = 5 mice in each in vivo experiment

Pertuzumab is a newly developed anti-HER2 antibody that targeting the CR I domain of HER2. Given the fact that HER2–103 covered most of the CR I sequence and its critical functions in TNBC, we proposed that HER2–103 positive TNBC maybe sensitive to Pertuzmab (Fig. 6a). We used MDA-MB-231 and MDA-MB-468 TNBC to generate mice xenografts and treated with Pertuzmab significantly reduced the tumor growth in vivo of both TNBC cells (Fig. 6b). Immunohistochemistry (IHC) of mice xenograft using p-EGFR and p-AKT antibody also supported that Pertuzumab dramatically inhibited the EGFR/Akt signaling activation in MDA-MB-231 and MDA-MB-468 TNBC (Fig. 6c). Furthermore, Pertuzmab treatment significantly reduced lung metastasis induced by tail-vein injection of MDA-MB-231 and MDA-MB-468 TNBC (Fig. 6d). In BT549 cells with low HER2–103 expression, Pertuzumab treatment did not altered the in vivo tumor growth or lung metastasis (Fig. 6b-d). Overexpression HER2–103 however, enhanced p-EGFR and p-AKT level in BT549 cells (Fig. 6c). Pertuzumab also inhibited HER2–103 overexpression induced xenograft growth and lung metastasis in BT549 cells (Fig. 6d).

## Discussion

We described that part of TNBC expressed circ-HER2, a circular RNA generated from *HER2* gene. Although HER2 mRNA was barely detected, circ-HER2 expression was identified in nearly 30% TNBC samples enrolled in this study and several established TNBC cell lines. Several reports addressed that circRNAs involved in proliferation or metastasis of TNBC; some circRNAs also had prognostic values [33]. However, current understanding of circRNAs in TNBC was limited to ‘microRNA sponge’, which absorbed micro RNAs and sequentially activated/inhibited downstream effectors [9, 34]. Ye et al. reported that circFBXW7 inhibited malignant progression of TNBC partly through encoding FBXW7-185aa [35], suggested additional layer of circRNAs’ function in this deadly cancer. Our discovery provided at least two ways of novel insight into TNBC. Firstly, as a splicing variant of *HER2* gene, circ-HER2 expression was enriched in parts of TNBCs. This finding demonstrated that certain TNBCs were not truly ‘HER2 negative’, as HER2–103 shared most of the amino acids as HER2 CR I domain. Secondly, HER2–103 played essential roles in those HER2–103 positive TNBCs. Knocking down HER2–103 by shRNA targeting circ-HER2 effectively attenuated the malignant phenotypes of TNBCs in vitro and in vivo. Given these results, circ-HER2 could serve as a ‘subtype indicator’ for some TNBCs. Indeed, circ-HER2 positive patients showed a worse total prognosis than those circ-HER2 negative patients in our 59 TNBC cohort. Due to the genetic amplification of *HER2* gene in HER2 positive breast cancers, HER2 mRNA and circ-HER2 were both significantly expressed (circ-HER2 was spliced from HER2 pre-mNRA), as well as their protein products HER2 and HER2–103. In TNBC, although HER2 protein was barely detected, considerable HER2–103 expression was found in certain tumors. This could be explained by that circ-HER2 had a considerably higher stability than HER2 mRNA.

Compensatory receptor tyrosine kinase (RTK) signaling such as EGFR, HER3 activation was observed in TNBC [36]. Specifically, EGFR is more frequently overexpressed in TNBC than other subtypes of breast cancer and has been recognized as a factor for poor prognosis [37]. However, targeting EGFR by TKIs or EGFR-antibody showed disappointing results in TNBC clinical trials, regardless of combination with chemotherapy or not [38, 39]. One of many possible explanations for EGFR-targeting therapy resistance of TNBC could be that HER3 upregulation and involved in the heterodimerization with EGFR [40]. This conclusion was supported by that abrogating HER3 upregulation in response to EGFR antagonist could inhibit the proliferation of TNBC cells and attenuates tumor growth in a mouse xenograft model [41]. Although combination of multiple RTK inhibitor showed promising effects in inhibition of TNBC under experimental condition, inevitable side-effects limited the clinical translation to human trials. Identification of more effective molecular targets in TNBC is desperately needed. In this study, we demonstrated that HER2–103 was required for EGFR/HER3 homo/heterodimer formation, phosphorylation and sequential AKT activation in certain TNBC. Deprivation HER2–103 in these TNBCs effectively attenuated p-EGFR kinase activities and p-AKT level, indicating that part of TNBCs could be targeted by HER2–103, a critical node for both EGFR/HER3 signaling network.

So far, Pertuzumab is only approved in HER2 positive breast cancers and usually used in combination with Trastuzumab [42]. Shared with the same antigen-recognition domain of HER2, HER2–103 also could be antagonized by Pertuzumab, indicated by our preclinical results in mice xenografts. In HER2–103 negative TNBCs, overexpressing HER2–103 induced tumoral progression also reversed by Pertuzumab, suggested the specificity. Our discovery of HER2–103 guaranteed future investigations: screening circ-HER2 expressed TNBC patients and verified the clinical effects of Pertuzumab by well-designed clinical trials. HER2–103 may provide a unique opportunity for those TNBC patients that expressing circ-HER2, whose only option is chemotherapy, to benefit from Pertuzumab.

## Conclusion

Circ-HER2 is a newly identified *HER2* transcriptional variant. Circ-HER2 encodes HER2–103. Circ-HER2 and HER2–103 are expressed in parts of TNBC, which could promote EGFR/HER3 interaction and activation. Deprivation HER2–103 inhibits the cells proliferation, invasion and tumorigenicity of TNBC in vitro and in vivo. Sharing the same amino acid sequences as the HER2 CR1domian, HER2–103 could be antagonized by Pertuzumab. Our discovery identified that circ-HER2/HER2–103 expressing TNBC patients could benefit from Pertuzumab, a clinically approved HER2 antibody.

## Supplementary information

**Additional file 1:** **Table S1.** All plasmids information.

**Additional file 2:** **Table S2.** 1200 differentially expressed circRNAs between 5 paired TNBC tissues.

**Additional file 3:** **Table S3.** 1609 differentially expressed host coding genes between 5 paired TNBC tissues.

**Additional file 4:** **Table S4.** 1425 host genes and 12 circRNAs that were contained in the darkred module.

**Additional file 5:** **Table S5.** 909 differentially expressed circRNAs derived from 697 host coding genes that were not differentially expressed between 5 paired TNBC samples.

**Additional file 6:** **Table S6.** All possible HER2 isoforms from NCBI.

**Additional file 7:** **Figure S1.** The 14 gene module constructed by weight gene network analysis (WGCNA). (**A**) Module Trees constructed by processing with WGCNA. (**B**) Number of genes in each module. (**C**) Heatmap of module-module relationship. Numbers in the heatmap were Pearson correlation coefficient between eigengene value of each modules and corresponding *p* value for coefficient (in brackets). (**D**) Eigengene expression pattern of each modules in the 10 samples. (**E**) Eigengene expression pattern of module darkred. (**F**) The 10 hub coding genes related to breast cancer in darkred module and circRNAs co-expressed with them with weight value > 0.15. The size of network points represents the connectivity of corresponding molecules in the module, and the thickness of lines represents the weight value of expression correlation between two molecules. The 5 circRNA were circ_NMRAL1 (circbase ID:hsa_circ_0007788), circ_HER2 (hsa_circ_0007766), circ_DLG1 (hsa_circ_0008500), circ_NSD2 (hsa_circ_001422), circ_CDYL(hsa_circ_0008285).

**Additional file 8:** **Figure S2.** Validation of translation potency of circ-HER2. (**A**) The putative IRES activity in circ-HER2 was tested. Left panel, IRES sequences in circ-HER2 or its different truncations/mutation were cloned between Rluc circular reporter genes. Right, different domians of IRES in circ-HER2 and the relative luciferase activity of Rluc in the above vectors was tested. ECMV IRES was used as positive control. (**B**) Circ-HER2 vector was transfected into MDA-MB-453 cells, which express low level of HER2 and circ-HER2.HER2–103 and HER2 level were decided by IB. The successful transfection was verified by q-PCR. (**C**) IB of concentrated cell culture suspensions from MDA-MB-231 and MDA-MB-468 with indicated modifications. Coomassie blue staining of total proteins was used as a loading control. The expression level of circ-HER2 RNA in the suspensions of these two TNBC cell lines were also detected. Lines show the mean ± SD, ***, *p* < 0.001; **, *p* < 0.01; *p < 0.05. Data are representative from at least 2–3 experiments with similar results.

**Additional file 9:** **Figure S3.** Circ-HER2 controls HER2 positive breast cancer cells tumorigecncity. (**A**) BT474 and SK-BR-3 HER2 positive breast cancer cells were stably transfected with scramble or circ-HER2 shRNAs. Circ-HER2 expression level and indicated protein levels were decided as indicated. (**B**) CCK-8 assay in BT474 and SK-BR-3 HER2 positive breast cancer cells transfected with scramble or circ-HER2 shRNA. (**C**) Soft agar colony formation assay in BT474 and SK-BR-3 HER2 positive breast cancer cells transfected with scramble or circ-HER2 shRNA. (**D**) Plate colony formation assay in BT474 and SK-BR-3 HER2 positive breast cancer cells transfected with control or circ-HER2 shRNA. (**E**) Transwell migration assays of BT474 and SK-BR-3 HER2 positive breast cancer cells transfected with control or circ-HER2 shRNA. Lines show the mean ± SD, ***, *p* < 0.001; **, *p* < 0.01; *p < 0.05. Data are representative from at least 2–3 experiments with similar results.

**Additional file 10:** **Figure S4.** HER2–103 enhanced HER2 signaling by interacting HER2. (**A**) HER2–103 was immunoprecipitated in BT474 and SK-BR-3 HER2 positive breast cancer cells, followed by immunoblotting with antibodies against HER2. (**B**) Left, HER2 contain six domains, namely, L1, CR1, L2, CR2, juxta-menbrane (JM) segment and internal cellular domain (ICD). Right, HER2–103-Flag was immunoprecipitated with anti-flag antibody. Coimmunoprecipitated truncated HA-tagged-HER2 was detected by anti-HA antibody. Data are representative from at least 2–3 experiments with similar results.

## Data Availability

Raw sequencing and processed RNA Seq data from this study have been deposited into NCBI SRP266211.

## Abbreviations

TNBC: Triple Negative Breast Cancer
RTK: Receptor tyrosine kinase
PI3K: Phosphoinositide 3-kinase
circRNA: circular RNA
Circ-HER2: Circular HER2
EGFR: Epidermal growth factor receptor
ORF: Open reading frame
IRES: Internal ribosomal entry site
WGCNA: Co-expression networks were constructed using
