# Supplementary figures and images for: Circular HER2 RNA positive triple negative breast cancer is sensitive to Pertuzumab
Source: Mol Cancer. 2020 Sep 11;19:142. doi: 10.1186/s12943-020-01259-6 (PMC7488427; doi:10.1186/s12943-020-01259-6)

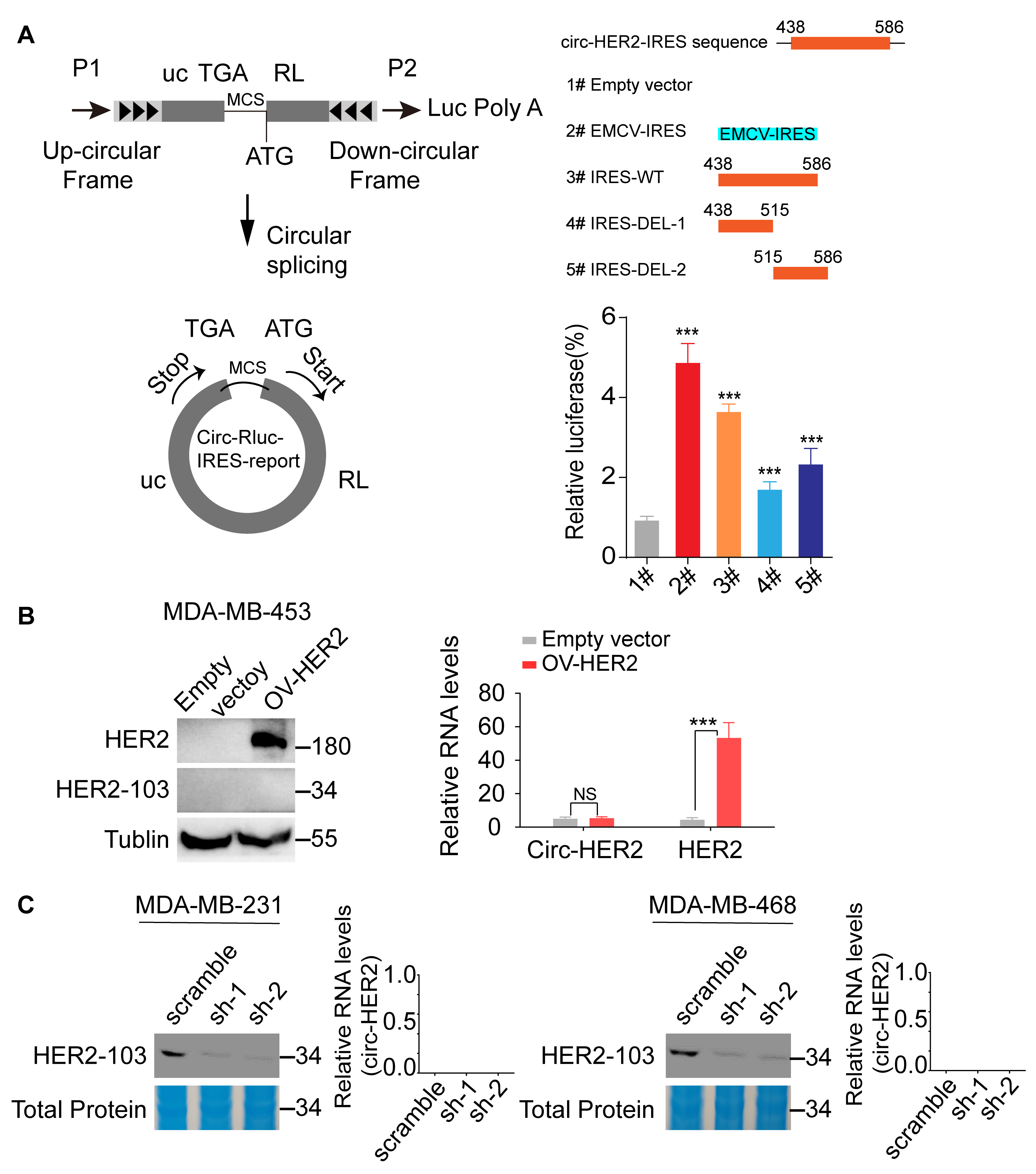

Supplement: Supplementary file 8 — Additional file 8: Figure S2. Validation of translation potency of circ-HER2. (A) The putative IRES activity in circ-HER2 was tested. Left panel, IRES sequences in circ-HER2 or its different truncations/mutation were cloned between Rluc circular reporter genes. Right, different domians of IRES in circ-HER2 and the relative luciferase activity of Rluc in the above vectors was tested. ECMV IRES was used as positive control. (B) Circ-HER2 vector was transfected into MDA-MB-453 cells, which express low level of HER2 and circ-HER2.HER2–103 and HER2 level were decided by IB. The successful transfection was verified by q-PCR. (C) IB of concentrated cell culture suspensions from MDA-MB-231 and MDA-MB-468 with indicated modifications. Coomassie blue staining of total proteins was used as a loading control. The expression level of circ-HER2 RNA in the suspensions of these two TNBC cell lines were also detected. Lines show the mean ± SD, ***, p < 0.001; **, p < 0.01; *p < 0.05. Data are representative from at least 2–3 experiments with similar results. [file 12943_2020_1259_MOESM8_ESM.tif]

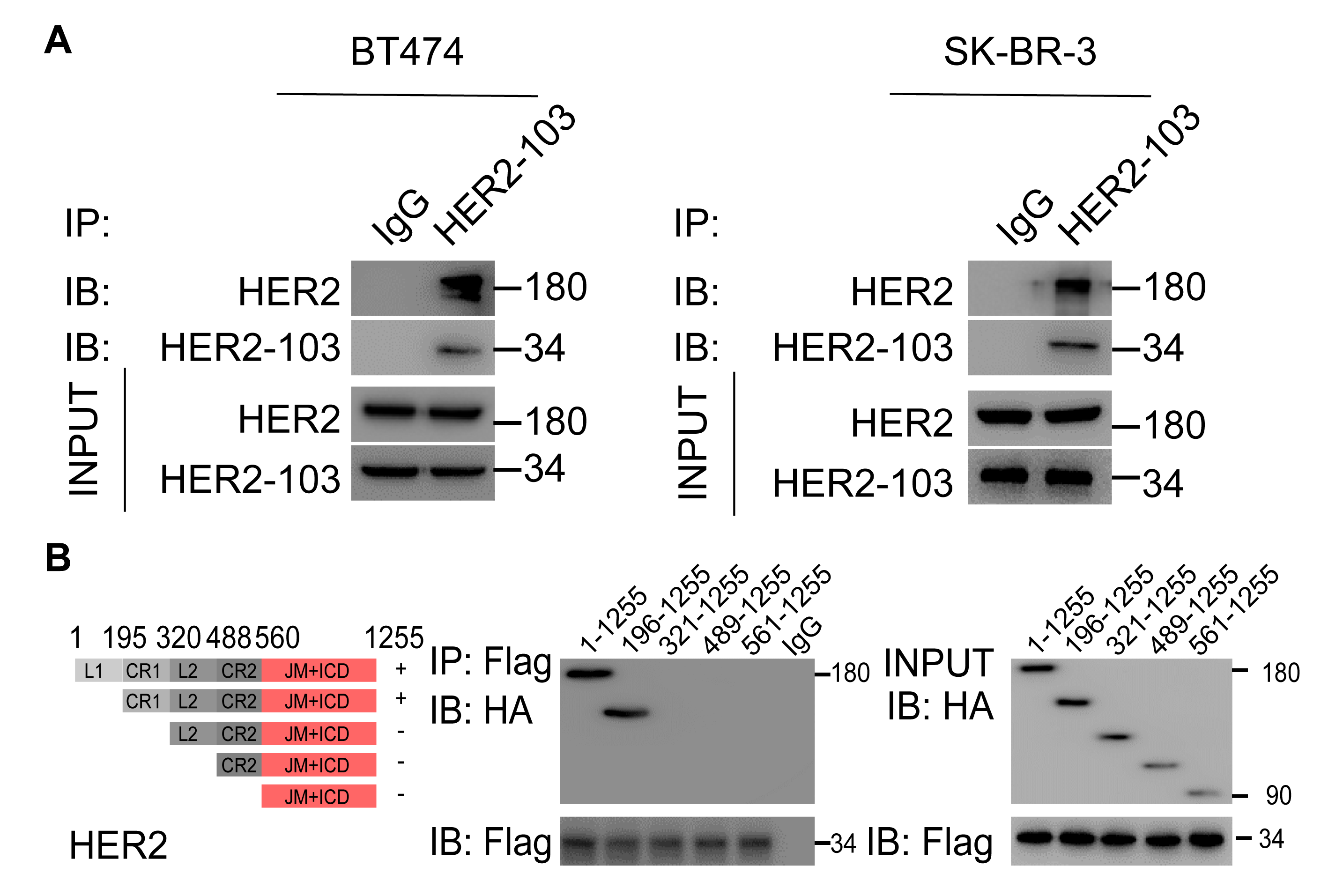

Supplement: Supplementary file 10 — Additional file 10: Figure S4. HER2–103 enhanced HER2 signaling by interacting HER2. (A) HER2–103 was immunoprecipitated in BT474 and SK-BR-3 HER2 positive breast cancer cells, followed by immunoblotting with antibodies against HER2. (B) Left, HER2 contain six domains, namely, L1, CR1, L2, CR2, juxta-menbrane (JM) segment and internal cellular domain (ICD). Right, HER2–103-Flag was immunoprecipitated with anti-flag antibody. Coimmunoprecipitated truncated HA-tagged-HER2 was detected by anti-HA antibody. Data are representative from at least 2–3 experiments with similar results. [file 12943_2020_1259_MOESM10_ESM.tif]
